# Lactylation in cancer: Mechanisms in tumour biology and therapeutic potentials

**DOI:** 10.1002/ctm2.70070

**Published:** 2024-10-25

**Authors:** Yipeng He, Tianbao Song, Jinzhuo Ning, Zefeng Wang, Zhen Yin, Pengcheng Jiang, Qin Yuan, Weimin Yu, Fan Cheng

**Affiliations:** ^1^ Department of Urology Renmin Hospital of Wuhan University Wuhan Hubei P.R. China

**Keywords:** clinical translation, lactylation, post translational modification, tumour biology, tumour microenvironment

## Abstract

Lactylation, a recently identified form of protein post‐translational modification (PTM), has emerged as a key player in cancer biology. The Warburg effect, a hallmark of tumour metabolism, underscores the significance of lactylation in cancer progression. By regulating gene transcription and protein function, lactylation facilitates metabolic reprogramming, enabling tumours to adapt to nutrient limitations and sustain rapid growth. Over the past decade, extensive research has revealed the intricate regulatory network underlying lactylation in tumours. Large‐scale sequencing and machine learning have confirmed the widespread occurrence of lactylation sites across the tumour proteome. Targeting lactylation enzymes or metabolic pathways has demonstrated promising anti‐tumour effects, highlighting the therapeutic potential of this modification. This review comprehensively explores the mechanisms of lactylation in cancer cells and the tumour microenvironment. We expound on the application of advanced omics technologies for target identification and data modelling within the lactylation field. Additionally, we summarise existing anti‐lactylation drugs and discuss their clinical implications. By providing a comprehensive overview of recent advancements, this review aims to stimulate innovative research and accelerate the translation of lactylation‐based therapies into clinical practice.

**Key points:**

- Lactylation significantly influences tumour metabolism and gene regulation, contributing to cancer progression.
- Advanced sequencing and machine learning reveal widespread lactylation sites in tumours.
- Targeting lactylation enzymes shows promise in enhancing anti‐tumour drug efficacy and overcoming chemotherapy resistance.
- This review outlines the clinical implications and future research directions of lactylation in oncology.

## Graphical abstract

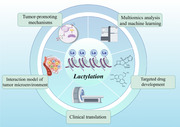

## 1 INTRODUCTION

### 1.1 Lactate and lactylation

It is now widely recognised that, within cells, glucose undergoes glycolytic metabolism, leading to the production of pyruvate. LDH is a tetrameric enzyme composed of either four LDHA or LDHB subunits.^1^ LDHA preferentially catalyses the conversion of pyruvate to lactate, while LDHB favours the reverse reaction.^2^ Despite LDHA knockdown, LDHB can compensate and generate lactate.^3^ Notably, lactic acid, with a pKa of 3.86, is predominantly present in its deprotonated form, lactate, under physiological conditions.^4^ In humans, L‐lactate is the principal form, with serum concentrations of 1–2 millimoles, while D‐lactate is present at nanomolar levels.^5^, ^6^ Beyond its traditional role as a metabolic waste product, lactate has emerged as a critical metabolite in various physiological processes. It has roles in regulating inflammation,^7^ promoting wound healing,^8^ exacerbating ischemia‐reperfusion injury,^9^ modulating the tumour microenvironment (TME)^10^, ^11^ and mediating the pathogenesis of various diseases.^12^, ^13^, ^14^ Monocarboxylate transporters (MCTs), such as MCT1 and MCT4, facilitate the transmembrane transport of lactate.^15^ The MCT‐mediated lactate shuttle has been implicated in diverse pathophysiological conditions.^16^, ^17^, ^18^, ^19^

Protein post‐translational modifications (PTMs) are a fundamental aspect of epigenetics. Common PTMs include methylation,^20^ acetylation,^21^ phosphorylation,^22^ ubiquitination,^23^ glycosylation,^24^ and succinylation.^25^ In 2019, Zhang et al. pioneered the identification and characterisation of histone lactylation in humans and mice using high‐performance liquid chromatography‐tandem mass spectrometry (HPLC‐MS/MS). Since then, lactate has become a significant player in epigenetic regulation.^26^ Histone lactylation reported by Zhang et al. involves the modification of lysine residues on histones by L‐lactate. Subsequent research has revealed that D‐lactate primarily targets non‐histone proteins, particularly glycolytic enzymes, where it regulates glycolysis through a negative feedback mechanism.^27^ Current evidence suggests that L‐lactate and D‐lactate exhibit distinct differences in substrate sources, target proteins and modification processes.

### 1.2 Lactate, tumour microenvironment and Warburg effect

The TME is a complex ecosystem composed of various cellular and non‐cellular components, including tumour cells, immune cells, blood vessels, stromal cells, and molecular signalling networks.^28^, ^29^, ^30^ The TME plays a critical role in tumour development and response to therapy. It influences the proliferation, invasion and metastasis of tumour cells.^28^, ^29^, ^31^ Therefore, understanding the composition and function of the TME, as well as its interactions with tumour cells, is of paramount importance for developing effective cancer treatments.

With the increasing understanding of the TME, the Warburg effect, originally proposed in 1927, has garnered renewed interest. While Warburg initially erred in positing aerobic glycolysis as the primary driver of tumourigenesis, it is now evident that this metabolic shift contributes to tumour progression.^32^ The core principle of the Warburg effect is that tumour cells preferentially utilise glycolysis for energy production, even in oxygen‐rich environments, rather than relying on oxidative phosphorylation. This metabolic switch results in the accumulation of substantial lactate, which not only serves as a metabolic by‐product but also functions as a crucial metabolic intermediate and signalling molecule, profoundly impacting the TME. Serum lactate levels in patients with tumours can range from 10 to 30 mM, with lactate concentrations within the tumour core often reaching 50 mM.^33^ Lactate produced by tumour cells in hypoxic regions, distant from blood vessels, is transported out of the cells via MCT4 and into the extracellular matrix. Conversely, tumour cells located near blood vessels, with relatively higher oxygen availability, can take up lactate from the matrix through MCT1 and oxidise it to generate ATP.^34^

As a substrate, lactate levels directly influence the extent of lactylation modifications, a relationship analogous to acetyl‐CoA and acetylation modifications.^35^ The TME provides abundant lactate, forming the basis for lactylation modifications in tumour and infiltrating cells.^11^, ^36^ Previous studies have demonstrated that lactate can influence immune cells in the tumour microenvironment, for example, by promoting the expression of CTLA‐4 in T cells,^37^ facilitating macrophage polarisation,^38^ and suppressing the immune activity of dendritic cells.^39^ Overall, lactate acts as a mediator of metabolic reprogramming in immune cells within tumours. Understanding this biological process is crucial for comprehending the mechanisms of tumourigenesis and development, as well as for innovating therapeutic strategies for cancer therapy.

### 1.3 Mechanisms of lactylation modification

**FIGURE 1:**
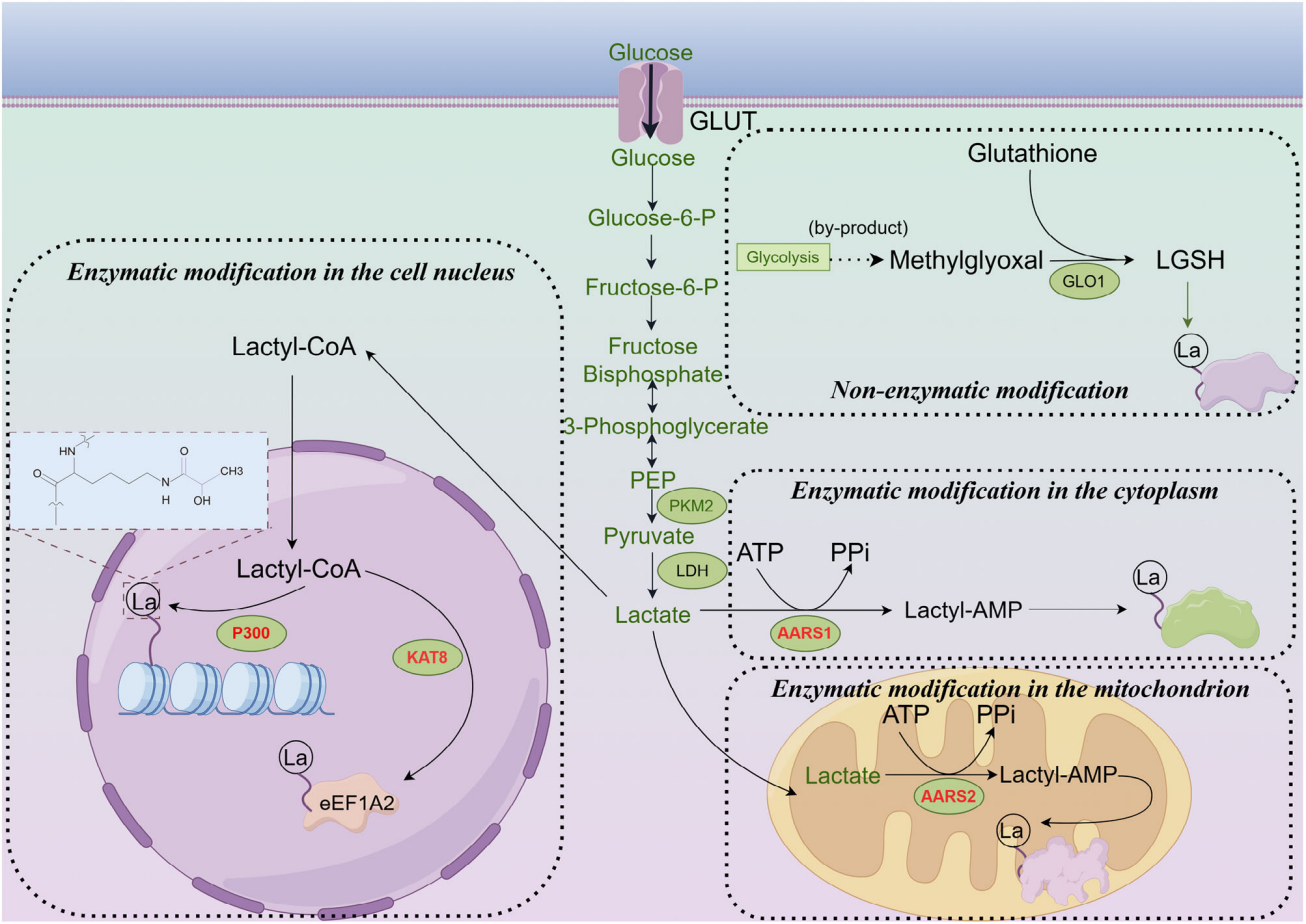
Four mechanisms of lactylation modification. The process of lactylation modification can be divided into enzymatic and non‐enzymatic reactions. Enzymatic reactions are mediated by different enzymes and can occur in the nucleus, cytoplasm and mitochondrial matrix. Non‐enzymatic reactions can take place in the cytoplasm.

Since its discovery, lactylation has garnered increasing attention. However, our understanding remains limited, particularly regarding substrates, specific enzymes and reaction kinetics. Currently, two primary mechanisms of lactylation are recognised: enzymatic and non‐enzymatic (Figure 1). Lactate, a common substrate for both pathways, exhibits different chiral forms. L‐lactate is involved in enzymatic lactylation, while D‐lactate participates in the non‐enzymatic process. Enzymatic lactylation involves two distinct pathways. The first pathway, proposed due to the similarity between lactylation and acetylation, entails the conversion of L‐lactate to L‐lactyl‐CoA, a direct substrate for lactylation. L‐lactyl‐CoA transfers the lactyl group to lysine residues on target proteins, a process catalysed by ‘writer’ enzymes.^26^ The second pathway, recently described in three independent studies, utilises aminoacyl‐tRNA synthetase 1 (AARS1) or AARS2. These enzymes directly convert lactate and ATP to lactate‐AMP, followed by the transfer of the lactyl group to lysine residues on substrate proteins, leading to lactylation modification.^40^, ^41^, ^42^ Non‐enzymatic lactylation involves D‐ lactate. Methylglyoxal (MGO), a by‐product of glycolysis, forms lactoylglutathione, which serves as the direct substrate for this non‐enzymatic process.^27^

Histone modifications are crucial components of epigenetic regulation. It is currently believed that histone lactylation primarily involves L‐ lactate. Consequently, studying the enzymes involved in this enzymatic process is essential. Most lactylation modifications involve writer and eraser enzymes. Due to the similarity between lactyl and acetyl groups, the first identified lactylation writer and eraser enzymes were those shared with acetylation. EP300^26^ and its homolog CREB‐binding protein (CBP)^43^ are lactylation writer proteins, while histone deacetylase 1–3 (HDAC) 1–3, HDAC8, and sirtuin (SIRT) 1–3 serve as the eraser proteins for this post‐translational modification.^44^, ^45^, ^46^ As research on lactylation advances, new lactylation enzymes have gradually been identified. Proteomic studies have revealed that upregulated lactylation in colorectal cancer correlates with poor prognosis, and lysine(K) acetyltransferase 8 (KAT8) was identified as a pan‐lactylation transferase for the first time. KAT8 enhances protein translation efficiency through lactylation of eEF1A2 at the K408 site, thereby promoting tumour progression.^47^ Similarly, KAT7 was later found to be a lactylation modification writing enzyme.^48^ Subsequently, AARS1^41^, ^42^ and AARS2^40^ were discovered; AARS1 primarily functions in the cytoplasm, while AARS2 is mainly localised in mitochondria. These enzymes utilise more direct substrates for lactylation. Given that lactyl‐CoA is almost undetectable in tumour cells and that its concentration is only 1/1000th that of acetyl‐CoA in cells, the efficiency of lactylation by acetyltransferases (such as p300) is significantly limited. Therefore, AARS1 and AARS2 are likely the primary writers of lactylation in tumour cells.^49^ However, the reaction efficiencies and functional differences of these enzymes have yet to be reported, and it is highly conceivable that many lactylation enzymes remain undiscovered. A summary of the currently reported lactylation writer and eraser enzymes is provided in Tables 1 and 2.

**TABLE 1:** Lactylation ‘writer’ enzymes.

Lactyltransferase	Subcellular location	Reaction substrate	Reference
EP300	Cell nucleus	Lactyl‐CoA	^26^
CBP	Cell nucleus	Lactyl‐CoA	^43^
KAT7	Cell nucleus	Lactyl‐CoA	^48^
KAT8	Cell nucleus	Lactyl‐CoA	^47^
AARS1	Cytoplasm	Lactate‐AMP	^41^, ^42^
AARS2	Mitochondrion matrix	Lactate‐AMP	^40^

**TABLE 2:** Lactylation ‘eraser’ enzymes.

Delactylases	Subcellular location	Reference
HDAC1	Cell nucleus	^46^
HDAC2	Cell nucleus and cytoplasm	^44^, ^46^
HDAC3	Cell nucleus and cytoplasm	^44^, ^46^
HDAC8	Cell nucleus and cytoplasm	^44^
SIRT1	Cell nucleus and cytoplasm	^50^
SIRT2	Cell nucleus and cytoplasm	^45^
SIRT3	Mitochondrion matrix	^44^

### 1.4 Factors affecting histone lactylation modification

Histone lactylation has been shown to enhance the transcription and expression of associated genes. Consequently, understanding the factors influencing histone lactylation is crucial in the field of epigenetic regulation. The biochemical reaction of lactylation modification involves substrates (lactyl‐CoA) and enzymes (writers and erasers) as the most direct influences. These substrates and enzymes within the biological signalling network are also affected by upstream regulatory points. These three elements constitute the key factors influencing histone lactylation modification.

1. Hypoxia and high lactate concentration: Lactate, produced by anaerobic respiration in cells, is the direct source of substrates for the lactylation modification reaction.^26^ In tumours, local hypoxia resolution leads to elevated lactate concentrations, which increase the extent of lactylation modification in their microenvironment.^51^ Similarly, local hypoxia generated during intense physical activity can also elevate the level of lactylation modification in cells.^40^
2. Lactylation modification enzymes: The writers (P300/KAT7/KAT8) and erasers (SIRT1‐3/HDAC1‐3/HDAC8) of histone lactylation modification determine the reaction rate. Changes in the quaternary structure of these enzymes can affect their enzymatic activity and ultimately regulate the level of histone lactylation modification. While various corresponding inhibitors^52^, ^53^, ^54^ have been reported for these enzymes, developing drugs that specifically regulate lactylation modification remains challenging due to their shared involvement in other acylation modifications. For instance, a previously reported semi‐synthetic derivative, LTK‐14A, has been shown to reduce butyrylation activity without affecting P300's acetylation activity, significantly decreasing intracellular H4K5 butyrylation.^55^
3. Upstream regulatory sites: Glycolysis is a pivotal upstream reaction for lactylation modification. Regulation of each step of glycolysis can influence the extent of lactylation modification.^56^ Additionally, the Hippo pathway^57^ and the Numb/Parkin pathway^58^ have been demonstrated to regulate the level of histone lactylation modification upstream.

### 1.5 Crosstalk between acetylation and lactylation modifications

Acetylation was one of the earliest identified acylation modifications of proteins, sharing similarities with lactylation in that both belong to the category of acylation modifications and utilise analogous writer and eraser proteins. However, they perform distinct functions within the cell. Both modifications are linked to acyl‐CoA as substrates, with acetylation being catalysed by the enzyme ACLY.^59^ The production process for lactyl‐CoA remains less well understood. The interaction and competition between these two modifications are complex and highly significant topics.

Both acetylation and lactylation tend to occur on lysine residues. When these modifications happen on histones, they often share sites, such as H3K18, H3K27 and H3K23,^26^ suggesting potential crosstalk between the two modifications. For example, macrophages, upon encountering bacterial stimuli, exhibit a gradual decrease in acetylation levels while lactylation levels increase.^26^ In hepatic stellate cells, a similar competition between acetylation and lactylation has been observed, where exogenous lactate promotes lactylation while suppressing acetylation.^60^ This process has been referred to as the ‘lactate clock’, where M1‐polarised macrophages shift toward M2 polarisation, thereby maintaining homeostasis. Additionally, the concentration of lactyl‐CoA in tumour cells is about 1/1000 of that of acetyl‐CoA.^49^ U‐13C6‐glucose incorporation kinetics also demonstrated that the time to reach a steady state for lactylation (24 h) was significantly shorter than that for acetylation (6 h).^26^ Taken together, the current evidence seems to suggest that, in most cases, lactylation is less competitive than acetylation.

The preferences of writer and eraser enzymes for these two modifications offer further insights into their relationship. p300 and CBP have been shown to exhibit differences in their specificity for acetylation substrates. In this respect, p300 demonstrates approximately ten times higher specificity than CBP for acetylating H3K14, H3K18 and H3K23. Conversely, CBP shows greater specificity, particularly for H3K18, when tetramers are used as substrates.^61^ However, the preference of p300 and CBP for lactylation versus acetylation remains unreported. HDACs have also been shown in some studies to exhibit clear preferences for both modification type and site. Class I HDAC inhibitors have been found to promote the acetylation of H3K18 in hepatic stellate cells while inhibiting lactylation at the same site.^60^ RNA interference of HDAC1 significantly increases lactylation at H4K5, whereas HDAC2 interference does not affect lactylation at this site, suggesting site‐specific regulation by HDACs.^46^

Notably, several HDAC inhibitors are already employed in clinical cancer therapy. For instance, panobinostat is indicated for the treatment of multiple myeloma,^62^ while romidepsin is used for T‐cell lymphoma.^63^ The effects of these two drugs on lactylation and acetylation in human patients remain a significant and worthwhile avenue for future investigation.

This review presents a comprehensive overview of recent advancements in the field of lactylation modifications, a burgeoning area of research in oncology. Drawing upon the latest frontier literature, this paper is the first to systematically categorise and summarise four distinct types of lactylation modifications. A comprehensive examination of existing research is provided, encompassing the mechanisms underlying tumour lactylation, machine learning‐based target prediction, drug development strategies, and the potential for clinical translation. Building upon this foundation, we propose promising avenues for future research in lactylation modifications. Ultimately, this review aims to contribute to the advancement of oncology by offering novel insights and perspectives that can inform future research endeavours and accelerate the clinical translation of lactylation‐related therapies.

## 2 MECHANISMS OF LACTYLATION MODIFICATION IN THE TME

The role of lactylation within the TME is a complex and multifaceted area of cancer biology. The TME, a dynamic and heterogeneous ecosystem composed of cancer cells, stromal cells, immune cells and the extracellular matrix, significantly impacts cancer growth and therapeutic responses. Within this environment, lactylation modulates diverse intercellular interactions and functions, suggesting novel avenues for understanding and targeting cancer.

### 2.1 Direct effects of lactylation modification on tumour cells

The tumour cell, the primary constituent of the TME, is both the instigator of elevated lactylation levels and the direct target of the Warburg effect. In most documented studies, increased lactylation within tumour cells has been strongly correlated with the intensification of their malignant phenotype.

**FIGURE 2:**
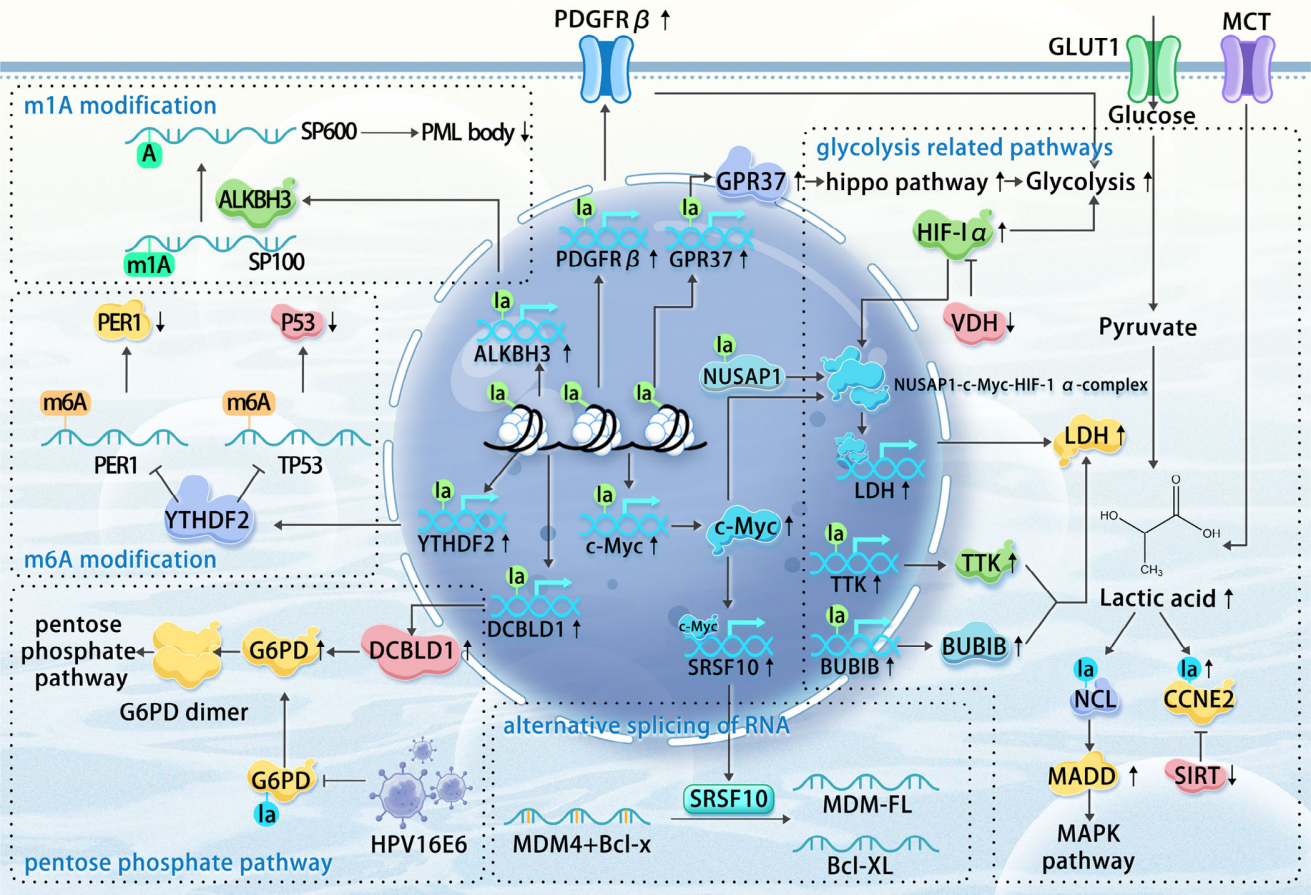
Pathways related to lactylation modifications in tumour cells. Extensive histone lactylation modifications within tumour cells drive the expression of various genes. The proteins corresponding to the expression of these genes activate downstream biological pathways in the cytoplasm, such as RNA alternative splicing and modification, as well as various classic signalling and metabolic pathways. Some of these proteins target glycolysis, leading to further increases in intracellular lactate levels, while others function as transcription factors that drive the expression of additional oncogenes. The metabolic network, derived from the core processes of glycolysis and lactylation modifications, elucidates the mechanisms underlying the malignant behaviour of tumour cells in a high‐lactate environment.

Lactylation modifications within tumour cells are intricate and multifaceted, involving complex signalling networks. Figure 2 provides a visual representation of the currently understood signalling pathways associated with lactylation modifications in tumour cells. Table 3 summarises the known lactylation targets and their corresponding signalling pathways in various tumour types. Lactylation modifications have been implicated in the progression of several cancers. For instance:

1. Bladder cancer: H3K18 lactylation enhances the expression of the oncogene lipocalin‐2 (LCN2), promoting tumourigenesis. However, the specific mechanisms by which LCN2 drives bladder cancer progression remain to be fully elucidated.^57^
2. Breast cancer: The accumulation of lactate, a by‐product of the Warburg effect, leads to H3K18la modification at the c‐Myc promoter. This upregulates serine and arginine‐rich splicing factor 10 (SRSF10) transcriptionally, which in turn regulates the alternative splicing of murine double minute 4 (MDM4) and Bcl‐x, ultimately promoting tumour progression.^64^
3. Cervical cancer: Lactate stabilises Discoidin, CUB and LCCL domain‐containing type I (DCBLD1) protein expression through lactylation, particularly at lysine 172. This inhibition of glucose‐6‐phosphate dehydrogenase (G6PD) autophagic degradation activates the pentose phosphate pathway (PPP), promoting cancer progression.^65^ Interestingly, G6PD itself is also a target for lactylation, with HPV16E6 promoting its delactylation and subsequent dimerisation, leading to PPP upregulation.^66^
4. Cholangiocarcinoma: Lactylation of nucleolin (NCL), particularly at lysine 477, regulates the RNA splicing of MAP kinase‐activating death domain protein (MADD), promoting intrahepatic cholangiocarcinoma progression by preventing nonsense‐mediated mRNA decay and increasing MADD expression.^67^
5. Clear cell renal cell carcinoma (ccRCC): VHL inactivation, a significant prognostic factor in ccRCC,^68^, ^69^, ^70^, ^71^ promotes platelet‐derived growth factor β (PDGFRβ) transcription through H3K18la, creating a positive feedback loop that enhances ccRCC development.^72^
6. Colorectal cancer: G protein‐coupled Receptor 37 (GPR37) activates the Hippo pathway, promoting lactate dehydrogenase A (LDHA) expression and glycolysis, ultimately promoting H3K18la and the upregulation of CXCL1 and CXCL5. GPR37 deficiency has been shown to inhibit liver metastasis in a mouse model.^73^
7. Endometrial cancer: Histone lactylation upregulates USP39 expression, which interacts with PGK1 to activate the PI3K/AKT/HIF‐1α signalling pathway and enhance glycolysis. Elevated lactate levels increase lactylation, creating a positive feedback loop.^74^
8. Esophageal cancer: Histone H3K9la promotes the transcription of LAMC2, facilitating cell proliferation and invasion.^75^
9. Glioma: XRCC1 lactylation at K247 enhances its nuclear translocation and DNA repair capabilities.^76^
10. Hepatocellular carcinoma (HCC): Low SIRT3 expression correlates with high levels of lactylation and poor prognosis. Lactylation of cyclin E2 (CCNE2) at lysine 348 enhances cancer cell invasiveness and proliferation.^77^
11. Ocular melanoma: Increased histone lactylation elevates YTH domain family protein 2 (YTHDF2) transcription, promoting the degradation of m6A‐modified PER1 and TP53 mRNAs, and accelerating tumourigenesis.^78^ Histone lactylation also removes m1A methylation from SP100A, enhancing AlkB homolog 3 (ALKBH3) expression and reducing the formation of tumour‐suppressive promyelocytic leukaemia (PML) bodies, thus promoting malignant transformation.^79^
12. Pancreatic ductal adenocarcinoma (PDAC): Nucleolar and spindle‐associated protein 1 (NUSAP1), a regulatory complex with c‐Myc and HIF1α, increasing LDHA expression and glycolysis, leading to elevated intracellular lactate levels. Lactylated NUSAP1 is more stable, contributing to a feedback loop exacerbating PDAC malignancy.^80^ The glycolysis‐H3K18la‐TTK/BUB1B positive feedback regulatory loop has also been shown to be involved in tumour development.^56^ P300‐mediated lactylation of nicotinamide nucleotide adenylyltransferase 1 (NMNAT1) at lysine 128 promotes its nuclear localisation and enzymatic activity, supporting the nuclear NAD salvage pathway and promoting PDAC progression.^81^ Lactylation of TFEB at the K91 site prevents its interaction with WWP2, inhibiting its ubiquitination and degradation, leading to increased TFEB activity and cellular autophagy.^82^
13. Prostate cancer: SIRT1‐mediated delactylation affects the cellular localisation of Canopy FGF signalling regulator 3 (CNPY3), promoting lysosomal rupture and triggering pyroptosis.^50^

**TABLE 3:** Sites of lactylation modification and related pathways.

Types of cancer	Lactylation site	Signalling pathway	Reference
Acute myeloid leukaemia	H3K18, H4K5, H4K8, H4K12	STAT5‐lactylation‐PD‐L1	^108^
Anaplastic thyroid cancer	H4K12	Lactylation‐CTGF, CCNE1, CDK1, KLF2, IL1B, AURKB	^109^
Bladder cancer	H3K18	circXRN2‐LATS1‐hippo‐lactylation‐LCN2	^57^
Breast cancer	K120 and K139 on p53	p53 pathway	^42^
Cervical cancer	K45 on G6PD	G6PD(delactylation)‐GP6D(dimer)	^66^
Cervical cancer	K172 on DCBLD1	DCBLD1‐G6PD‐pentose phosphate pathway	^65^
Clear cell renal cell carcinoma	H3K18	Histone lactylation‐PDGFRβ signalling positive feedback loop	^72^
Colorectal cancer	H3K18	GPR37‐LATS1‐YP1‐lactylation‐CXCL1/CXCL5	^73^
Colorectal cancer	H4K12	SMC4‐diapuse like state‐glycolysis enzymes‐lactylation	^110^
Colorectal cancer	H3K18	Lactylation‐RUBCNL	^111^
Colorectal cancer	Pan‐lactylation	PCSK9‐lactylation‐M2 polarisation	^83^
Colorectal cancer	H3K18	Lactylation‐RARγ‐NF‐kB‐IL‐6	^84^
Colorectal cancer	K408 on eEF1A2	KAT8‐eEF1A2	^47^
Endometrial carcinoma	H3K18	Kla‐USP39‐PGK1‐PI3K/AKT/HIF‐1α signalling pathway	^74^
Gastric cancer	K90 on YAP, K108 on TEAD1	Hippo pathway	^41^
Glioblastoma	H3K18	Lactylation‐CD39, CD73, CCR8	^85^
Glioblastoma	H3K18	Lactate‐lactylation‐MAP4K4‐JNK pathway	^112^
Liver cancer	K348 on CCNE2	SIRT3‐CCNE2	^77^
Liver cancer	K72 on MOESIN	MOESIN‐TGFb pathway‐SMAD‐FOXP3	^74^
Liver cancer	H3K9, H3K56	–	^113^
Lung/prostate cancer	H3K18	Numb/Parkin‐lactylation	^58^
Ocular melanoma	H3K18	Lactylation‐YTHDF2 ‐YP53/PER1	^78^
Pancreatic adenocarcinoma	K128 on NMNAT1	NMNAT1‐NAD salvage pathway	^81^
Prostate cancer	H3K18	Lactylation‐HIFa‐sema3A	^114^
Prostate cancer	K215 and K224 on CNPY3	CNPY3‐pyroptosis	^50^

While most existing research suggests a pro‐oncogenic role for lactylation in tumours, it is crucial to acknowledge the possibility of tumour‐suppressive functions in certain contexts. Variations in histone lactylation levels across different cancer types can lead to distinct gene expression profiles, influencing cellular development and fate. Furthermore, lactylation of non‐histone proteins may exhibit varying functionalities under different biological conditions. Therefore, further targeted research is essential to fully elucidate the specific roles of lactylation and its associated proteins in various cancers, as well as the underlying molecular mechanisms involved.

Lactylation within tumour cells plays a pivotal role in shaping the abnormal metabolic landscape of the TME. Beyond its influence on metabolism, lactylation directly contributes to the progression of various cancer types through intricate mechanisms. The modification of histones and other key proteins by lactylation represents critical regulatory nodes that govern diverse malignant behaviours of tumours. A deeper understanding of the molecular mechanisms underlying lactylation in tumour cells offers novel insights into the interplay between metabolic reprogramming and epigenetic alterations in cancer. This knowledge provides a theoretical foundation for the development of innovative anticancer strategies targeting lactylation‐related pathways.

### 2.2 Lactylation modification and immunosuppressive immune microenvironment

**FIGURE 3:**
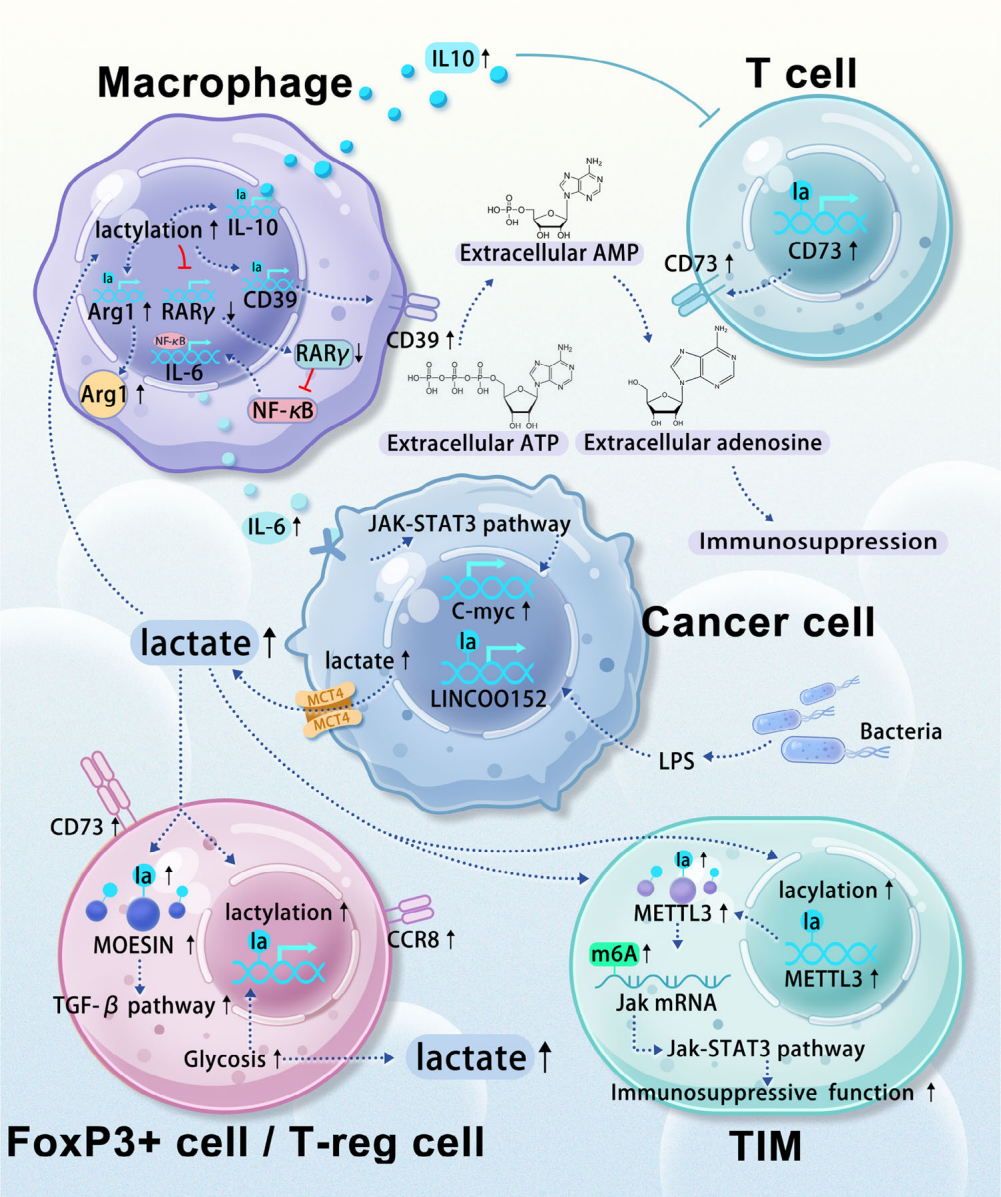
Relevant pathways of lactylation modifications in the tumour microenvironment. The increase in lactate within the TME leads to the lactylation of infiltrating cell proteins, ultimately assisting the tumour in adapting to and exploiting the microenvironment. Tumour cells upregulate glycolysis, exporting large amounts of lactate into the microenvironment. This elevated lactate level in infiltrating cells subsequently increases the lactylation levels of their intracellular proteins. Across various immune cell types, including macrophages, T cells, T‐reg cells, or tumour‐infiltrating myeloid (TIM) cells, the upregulation of intracellular lactylation is associated with a shift towards immunosuppressive and pro‐tumour phenotypes ultimately promoting tumour survival and proliferation.

The immune cells infiltrating the TME are a key focus of cancer research. The accumulation of lactate within the TME has been linked to immune response suppression, enabling cancer cells to evade immune detection. Lactylation modifications alter the metabolic status and signalling pathways of various immune cells, affecting their function (Figure 3). A comprehensive understanding and targeted intervention of these lactylation‐induced changes could potentially enhance the efficacy of immunotherapy.

Lactylation modifications in tumour‐associated cells can promote the transition of immune cells toward an immunosuppressive phenotype. Macrophages, key players in the immune response, exist in two primary phenotypes: M1 and M2. M1 macrophages are involved in acute immune reactions, while M2 macrophages are typically associated with parasitic diseases. Within the TME, macrophages often exhibit an M2 phenotype characterised by immunosuppression. Recent studies have highlighted a strong correlation between histone lactylation modification in tumour‐associated macrophages (TAMs) and their cellular fate, with higher lactylation levels favouring the M2 phenotype.^26^, ^83^ In colorectal cancer, tumour‐derived lactate promotes H3K18 lactylation in macrophages, suppressing RARγ gene transcription and increasing interleukin‐6 (IL‐6)levels in the TME. This subsequently activates the STAT3 signalling pathway in CRC cells, promoting c‐Myc gene transcription and the transformation of macrophages into an M2‐like pro‐tumour phenotype.^84^ In glioma microenvironments, elevated lactate levels have been shown to increase the lactylation levels of various genes, remodelling them into pro‐tumour phenotypes. Upregulated genes include CD73 in tumour cells, CD39 and CCR8 in T‐reg cells, CD39 in macrophages, and CD73 in T cells.^85^ Extracellular ATP is converted to AMP by CD39 and then to adenosine by CD73.^86^ Adenosine and extracellular ATP further remodel the microenvironment into an immunosuppressive landscape.^87^, ^88^, ^89^, ^90^ Additionally, the elevated lactylation modification of macrophage histones in the TME increases the expression of IL‐10, a cytokine that induces T cell immune silence.^91^

Lactylation modifications in the tumour microenvironment can also promote the generation of immunosuppressive cells. FOXP3^+^ and T‐reg cells are potent immunosuppressors within this environment.^92^, ^93^, ^94^ Prolonged lactate accumulation has been shown to induce the upregulation of H3K18 lactylation levels in NKT cells, leading to the generation of FOXP3^+^ NKT cells. Additionally, the widespread upregulation of the glycolytic pathway in these cells further promotes lactylation within the entire microenvironment.^95^ In vitro experiments have demonstrated that a high lactate environment favours the stability of T‐reg cells, while lactate degradation reduces their induction. Specifically, a high lactate environment leads to an increase in the lactylation of lysine 72 of MOESIN within these cells. Lactylated MOESIN enhances the effects of the TGF‐β pathway, promoting the stability and generation of these immunosuppressive cells.^96^

Lactylation modifications in the tumour microenvironment can enhance the function of immunosuppressive cells. Accumulated lactate induces the upregulation of methyltransferase‐like 3 (METTL3) in tumour‐infiltrating myeloid cells (TIMs) through H3K18 lactylation. Two lactylation modification sites within the zinc finger domain of METTL3 are crucial for RNA capture efficiency. The downstream METTL3‐JAK1‐STAT3 regulatory axis promotes the immunosuppressive function of TIM.^97^

Fibroblasts constitute a significant portion of the stromal cells within the TME,^98^ contributing substantially to the high levels of lactate present.^99^ Lactate, in turn, is directly responsible for the increased level of lactylation modifications in infiltrating cells within the microenvironment. In pancreatic cancer, tumour‐associated fibroblasts can utilise lactate to maintain a fibrotic and immunosuppressive microenvironment.^100^ In prostate cancer, lactate secreted by fibroblasts has been shown to inhibit the activity of CD4 T cells.^101^ However, the specific mechanisms by which these predominantly stromal cells interact with immune cells via lactylation modifications remain unclear, and future research should focus on this area.

In summary, lactylation modifications in the TME remodel the immune microenvironment through three primary mechanisms: promoting the transition of immune cells towards an immunosuppressive phenotype, promoting the generation of immunosuppressive cells, and enhancing the function of immunosuppressive cells. A comprehensive understanding of these pathways and the development of targeted therapeutic approaches are essential for reversing the ‘cold’ tumour microenvironment and improving the efficacy of immunotherapy.

### 2.3 Bacteria and lactylation modification

The evolving understanding of the TME reveals the presence of bacteria in various tissue tumours. Early studies primarily focused on bacteria in mucosal‐related carcinomas, such as those affecting the skin,^102^ lungs^103^ and gastrointestinal tract.^104^, ^105^ However, recent findings, including the identification of intracellular bacteria in breast cancer, have demonstrated the widespread presence of bacteria in non‐mucosal tumour microenvironments.^106^ These findings challenge previous assumptions and highlight the need for further investigation of microbial dynamics across diverse tumour types.

Recent advancements in colorectal cancer research have revealed intricate connections between bacteria and epigenetic regulatory mechanisms. Lipopolysaccharides (LPS), components of bacterial cell walls, influence host gene expression. Specifically, LPS enhances the expression of LINC00152, a long noncoding RNA, by facilitating lactylation modifications within its promoter region, thereby increasing colorectal cancer's migratory and invasive capabilities.^107^ While the precise mechanisms underlying LPS's impact on these epigenetic modifications warrant further investigation, exploring the relationship between lactylation modifications and bacteria in other tumour contexts with established bacterial presence presents a promising avenue for future research.

## 3 MACHINE LEARNING AND LACTYLATION IN CANCER PROGNOSTICS: A DEEP DIVE INTO ADVANCED ANALYTICS

While recent foundational research has identified numerous lactylation modification sites, machine learning has significantly enhanced our ability to discover and exploit these sites. By integrating machine learning into the study of lactylation in cancer, researchers can more effectively understand and predict the progression and treatment of this complex disease. The sophisticated analytical capabilities of machine learning tools allow for the analysis of large datasets, revealing intricate patterns of lactylation that can influence tumour behaviour and patient prognosis.

### 3.1 Biomarker identification

Machine learning has been instrumental in identifying novel biomarkers associated with lactylation, offering potential drug targets. By analysing large datasets of gene expression and protein modification patterns, these models can pinpoint specific lactylation sites that are critical for tumour development and maintenance. These sites can subsequently serve as targets for the development of new drugs, opening avenues for innovative therapeutic approaches that disrupt key lactylation‐dependent pathways in cancer cells.

Recent proteomic studies have identified numerous lactylation modification sites in various cancer types. Hong et al. utilised machine learning and proteomic techniques to identify 2045 lactylation modification sites on 960 proteins in HCC.^115^ While the biological significance of these sites awaits further exploration, this proteomic investigation provides a solid foundation for studying protein lactylation modification in liver cancer. Similarly, in hepatitis B virus‐related hepatocellular carcinoma, 9256 non‐histone lactylation modification sites and 19 histone lactylation modification sites were identified. Additionally, through liquid chromatography‐tandem mass spectrometry (LC‐MS/MS) analysis and machine learning, 2735 high‐confidence lactylation modification sites were discovered in 1014 proteins in gastric cancer tissues. Interestingly, GO and KEGG analyses revealed that lysine lactylation modification proteins are mainly enriched in spliceosome‐related functions, providing new insights into the interaction between lactylation modification and other epigenetic modifications.^116^ Furthermore, using mass spectrometry techniques, researchers identified protein lactylation in SW480 colon cancer cells, identifying 637 lysine lactylation sites in 444 proteins. Of note, phosphofructokinase (PFKP) was lactylated at lysine 688, which was found to weaken its enzyme activity, thereby affecting glycolysis.^117^ This study suggests lactylation may constitute a lactate‐mediated negative feedback regulatory mechanism controlling metabolic reprogramming in tumour cells. The level of lactylation modification at H3K18 has been proven to be a risk factor for prognosis in ovarian cancer,^118^ and lactylation modifications have been shown to correlate with Bcl‐2, C‐myc, and P53 levels in lymphomas.^119^ Proteomics sequencing of lactylation in gastrointestinal tumours has further revealed that the lactylation of CBX3 at the K10 site can promote tumour progression.^120^

Overall, future research should focus on identifying and characterising the ‘key’ modification sites that regulate cell metabolism and determine cell fate. Additionally, investigating the biological pathways and functions enriched within the vast regulatory network of modification sites is a promising area of exploration. Finally, developing drugs targeting lactylation modification sites based on a comprehensive understanding of these sites holds significant translational potential.

### 3.2 Predictive modelling and personalised medicine

Utilising known lactylation modifications, researchers have developed machine learning prognostic models. These models offer the potential for personalised medicine by predicting tumour behaviour based on lactylation modification features. By estimating patient prognosis and identifying appropriate treatments, these models can guide clinicians in making informed decisions. This approach goes beyond one‐size‐fits‐all strategies and can contribute to the effective use of targeted therapy and immunotherapy, which are most effective for patients with specific tumour characteristics. Machine learning can analyse patients' transcriptomic features to predict their response to these therapies, enabling more personalised treatment approaches.

Several prognostic models have been developed based on lactylation modification target genes in various cancers, including hepatocellular carcinoma,^121^ gastric cancer,^122^ osteosarcoma,^123^ breast cancer^124^ and prostate cancer.^125^ Additionally, models have been constructed using lactylation ‘writer’ and ‘eraser’ proteins in hepatocellular carcinoma.^126^ N6‐methyladenosine (m6A) is a methylation modification that occurs on RNA.^127^ Recent studies have found that lactylation modification drives the expression of METTL3 and YTHDF, confirming a relationship between these two modifications.^78^, ^97^ Due to the demonstrated biological connection between lactylation modification and m6A modification, prognostic models based on genes related to both epigenetic modifications have shown satisfactory predictive ability.^128^ These models often utilise bulk RNA‐seq to screen for lactylation‐related genes and employ lasso‐cox regression to construct predictive models. Bioinformatics analyses, such as ssGSEA and pRRophetic, can be used to compare high‐ and low‐risk groups based on the models, assessing factors like immune infiltration, tumour mutation, and drug sensitivity. Such models consider the heterogeneity of cancer in lactylation modification biological processes among different patients, providing a personalised approach to treatment.

Prognostic models based on lactylation modification‐related genes may not be the only ones connected to lactylation. A prognostic model based on the solute carrier family gene SLC25A29 has shown lactylation modifications at the H3K14 and H3K18 sites of its promoter.^129^ Given the prevalence of high lactate concentrations in tumour microenvironments, various tumour prognostic models based on deep learning may also have biological significance related to lactylation modification. Further investigation into whether there are differences in lactylation modification levels between high‐ and low‐risk groups defined by other models (non‐lactylation modification‐related models) could provide valuable insights.

To further advance our understanding of lactylation modifications in cancer and develop more reliable models for diagnosis and treatment, it is essential to acquire larger‐scale multi‐omics data, particularly proteomics data. Such data can be used to train machine learning models to provide personalised treatment recommendations based on a comprehensive analysis of each patient's tumour characteristics.

## 4 THERAPEUTIC POTENTIAL OF TARGETING LACTYLATION: EXPANDING HORIZONS IN CANCER TREATMENT

**FIGURE 4:**
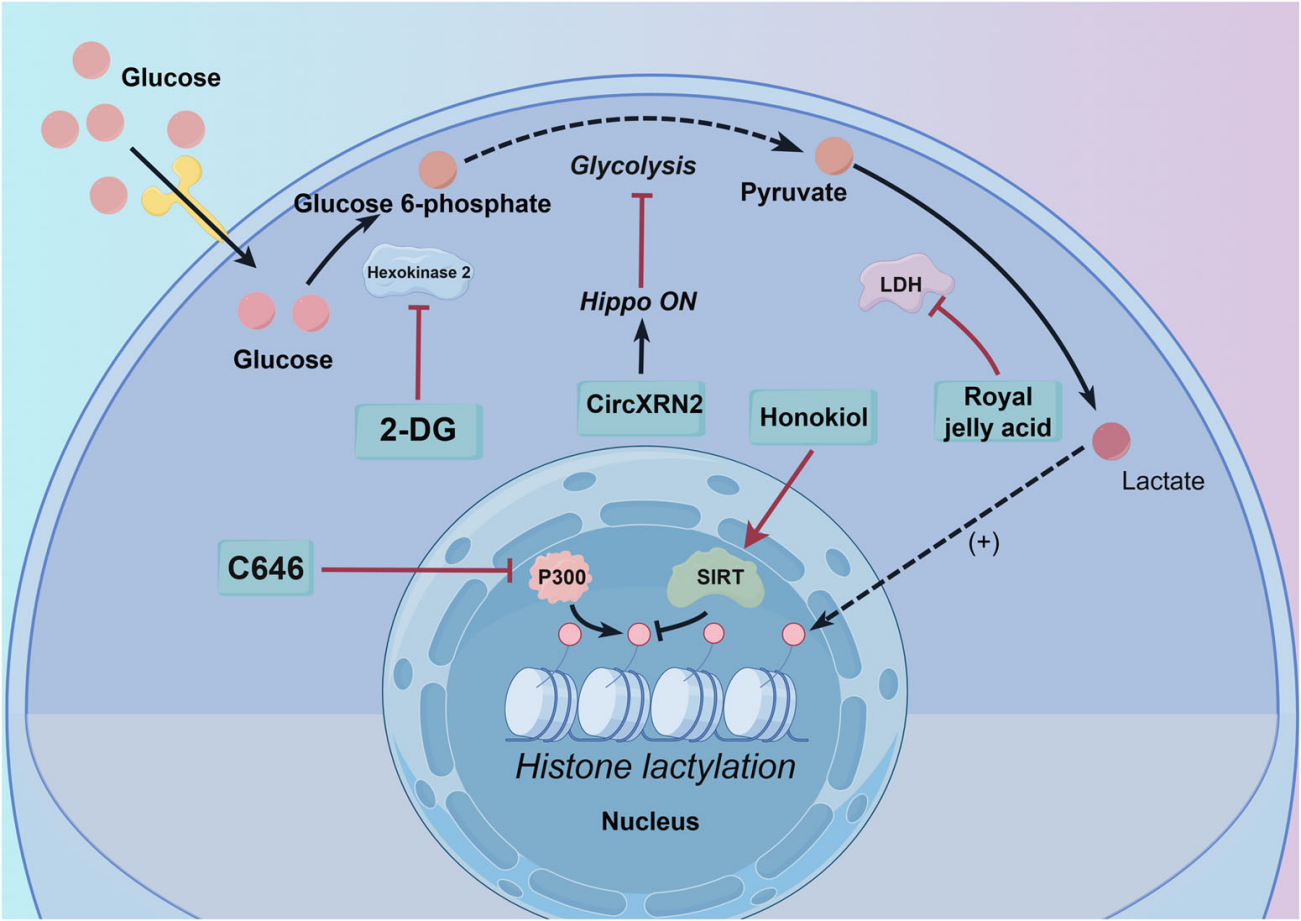
Different drugs inhibiting the glycolysis‐lactylation pathway. Different substances inhibit the glycolysis‐lactylation pathway. 2‐DG inhibits hexokinase, thereby preventing the conversion of glucose to glucose‐6‐phosphate. CircXRN2 has been shown to inhibit the glycolysis pathway. Royal jelly acid inhibits LDH, thus preventing the conversion of pyruvate to lactate. C646 inhibits the histone lactylation writer enzyme p300. Honokiol promotes the activity of the histone modification eraser enzyme SIRT.

A deeper understanding of lactylation mechanisms and targets in tumours has facilitated the investigation of related targeted therapies. Understanding how lactylation influences tumour cells at the molecular level is crucial for developing novel treatment strategies. Figure 4 illustrates known inhibitors of lactylation. These inhibitors either directly target the lactylation process or indirectly inhibit it by targeting upstream metabolic pathways.

### 4.1 Direct targeting of lactylation enzymes

One of the primary approaches to targeting lactylation involves modulating the enzymes associated with this modification. Lactyltransferases, the ‘writers’ of lactylation, are key players in the process. By targeting these enzymes or the ‘erasers’ of lactylation, such as SIRT enzymes, intracellular lactylation levels can be regulated. C646, an inhibitor of the lactylation writer enzyme p300, has been shown to synergise with the targeted drug PLX4032 (also known as Vemurafenib, a Raf inhibitor), demonstrating enhanced anti‐tumour effects compared to either drug alone.^109^ Many HDAC inhibitors, including Trichostatin A and Apicidin, have been reported to increase global lactylation levels, while TMP195 has no effect.^46^ However, the impact of HDAC inhibitors on lactylation can be complex, as they may promote acetylation, a competing modification. Honokiol, a SIRT3 activator, has been shown to possess anti‐tumour capabilities in hepatocellular carcinoma through its direct activation of delactylation enzymes.^77^ Numerous drugs targeting HDAC and SIRT regulation are currently under investigation, and assessing their impact on lactylation modification remains an important area of research.

### 4.2 Targeting metabolic pathways related to lactylation modification

Cancer cells often exhibit altered metabolic characteristics, characterised by increased glycolysis and lactate production. This metabolic shift, known as the Warburg effect, not only provides energy for rapid cell growth but also creates an environment conducive to lactylation. Consequently, targeting cancer metabolism offers an indirect strategy to modulate lactylation. Such an approach may involve drugs that inhibit glycolysis or regulate other metabolic pathways, potentially reducing the levels of lactylation modifications.

Modulating glycolysis through therapies like royal jelly acid can inhibit tumour growth by influencing lactylation modification pathways. Royal jelly is a substance secreted by the hypopharyngeal and mandibular glands of worker bees (*Apis mellifera*).^130^ Royal jelly acid (10‐hydroxy‐2‐decenoic acid) has been shown to suppress glycolysis‐related metabolic pathways in liver tumour cells, reducing lactylation modifications at the H3K9la and H3K14la sites through inhibition of lactate dehydrogenase.^131^ Targeting glycolytic pathways can also enhance the efficacy of existing therapies. For instance, 2‐DG, a hexokinase inhibitor, can increase the sensitivity of thyroid cancer to targeted therapy.^109^ As lactate is the direct substrate for lactylation modification, inhibiting LDH can reduce lactylation levels by lowering intracellular lactate concentrations. Oxamate, a competitive LDH inhibitor, has been widely demonstrated to decrease lactylation levels.^26^, ^56^

Beyond traditional metabolic pathway inhibitors, emerging therapeutic strategies target lactylation modifications to achieve anti‐tumour effects. Circular RNAs (circRNAs), a class of endogenous non‐coding RNAs, play diverse roles in various diseases.^132^, ^133^, ^134^ CircXRN2, for instance, activates the Hippo signalling pathway by interacting with the large tumour suppressor homolog 1 (LATS1) protein, thereby suppressing tumour growth through the inhibition of H3K18la and downregulation of LCN2 expression.^57^ While circRNAs are generally considered oncogenic factors in the urinary system, their abnormal expression in vivo is strongly linked to the development of urinary system tumours, making them potential biomarkers for diagnosis and prognosis.^135^ Recent research has also revealed that lactate, released by KRAS‐mutant tumour cells, promotes histone lactylation, directly facilitating the transcriptional activation of CircATXN7. CircATXN7 subsequently binds to the P65 subunit of the NF‐kB complex, masking its nuclear localisation signal and preventing its entry into the nucleus, effectively sequestering P65 in the cytoplasm. High expression of CircATXN7 in cytotoxic T lymphocytes (CTLs) is associated with poor prognosis and resistance to immunotherapy.^136^ In conclusion, while the role of circRNAs in cancer and lactylation modifications remains complex, lactylation modifications offer promising avenues for exploring the potential anti‐tumour effects of these conserved non‐coding RNAs.

## 5 CLINICAL AND RESEARCH IMPLICATIONS OF LACTYLATION IN CANCER

**FIGURE 5:**
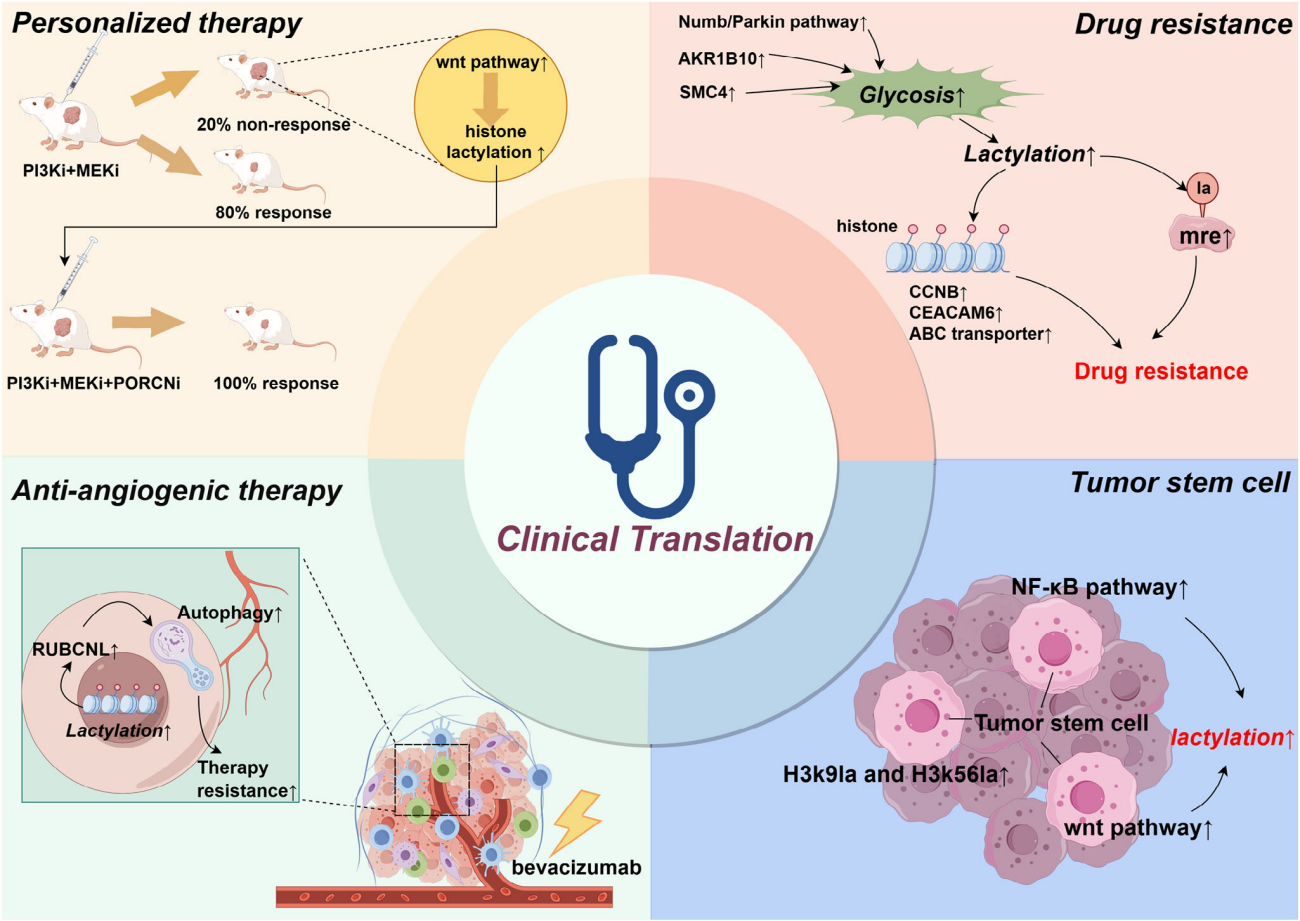
Four potential directions for clinical translation. Currently, there are four possible directions for the clinical translation of lactylation modification: personalised therapy, chemoresistance, anti‐angiogenic therapy and cancer stem cells. In personalised therapy, the addition of Wnt inhibitors to PI3Ki+MEKi therapy in non‐responsive mice has been shown to inhibit downstream lactylation modification, achieving a 100% response rate in these previously non‐responsive mice. Regarding chemoresistance, the upregulation of various signalling molecules activates the glycolysis‐lactylation axis. Both histone and non‐histone lactylation modifications downstream enhance tumour resistance to chemotherapy. In anti‐angiogenic therapy, the use of bevacizumab leads to increased tumour lactylation modification and elevated autophagy levels. For cancer stem cells, lactylation modifications induced by multiple signalling pathways contribute to enhanced tumour stemness.

Developing therapies that directly target lactylation modifications and related processes remains a challenging endeavour. In the meantime, a more clinically feasible approach is to examine the interplay between existing therapies and lactylation modifications. The aim is to mitigate the adverse effects of lactylation modifications on current cancer treatments, thereby optimising existing therapies and enhancing their anti‐tumour efficacy. Figure 5 outlines the four potential clinical translation directions that will be discussed below.

### 5.1 Personalised medicine and immunotherapy

Personalised medicine, which tailors treatment plans to the unique characteristics of each patient's disease, can significantly benefit from lactylation research. By understanding tumour‐specific lactylation profiles and implementing targeted therapies, clinicians can predict tumour responses to various treatments and select the most effective options for patients. This approach is especially crucial for immunotherapy, which achieves optimal outcomes when matched to specific molecular features of tumours.^137^, ^138^ The lactate‐rich tumour microenvironment, a consequence of the Warburg effect, profoundly influences tumour progression and immune evasion. High lactate concentrations within the microenvironment may lead to increased lactylation,^139^ affecting both tumour and immune cells. Consequently, modulating lactylation within the tumour microenvironment could potentially enhance the efficacy of immunotherapy.

Individual patient differences significantly contribute to the low response rates of some patients to immunotherapy.^140^, ^141^, ^142^, ^143^ Evidence suggests that liver cancer patients who respond to PD‐1 therapy exhibit significantly lower levels of MOESIN lactylation compared to non‐responders. In mouse models, combining anti‐PD‐1 therapy with lactate dehydrogenase inhibitors resulted in significantly smaller tumour volumes than PD‐1 therapy alone.^96^ In newly diagnosed acute myeloid leukaemia (AML) patients, bone marrow lactate levels positively correlate with STAT molecule expression, lactylation levels in cancer cells, and PD‐L1 expression, suggesting that AML patients with high bone marrow lactate levels might benefit more from immunotherapy.^108^ Moreover, recent studies demonstrate that targeting lactylation modifications is an effective strategy against TAMs. These macrophages undergo metabolic reprogramming by tumour cells, transforming into an immunosuppressive M2 phenotype.^144^, ^145^ Research shows that lactylation modification sites are present in TAMs and that lactylation levels are associated with the macrophage shift to the M2 phenotype.^26^ Combined treatment with PI3K and MEK inhibitors controls tumour growth in 80% of PTEN/p53‐deficient prostate cancer mice by inhibiting histone lactylation (H3K18la) in TAMs. In the remaining 20% of non‐responsive mice, feedback activation of the Wnt/β‐catenin pathway was observed, leading to the restoration of H3K18la and suppression of macrophage immune activity. Adding Wnt pathway inhibitors subsequently resulted in a 100% remission rate in prostate cancer mice.^146^

The TME contains various immunosuppressive immune cell phenotypes, including myeloid‐derived suppressor and regulatory T cells. Targeting lactylation modifications could potentially exert positive anti‐tumour effects by influencing these cells. In conclusion, therapies targeting lactylation modifications may help reverse the immunosuppressive ‘cold’ tumour microenvironment in patients who are unresponsive to immunotherapy, thereby improving response rates to immunotherapy through personalised treatment strategies.

### 5.2 Lactylation and drug resistance

One of the primary challenges encountered in cancer treatment is the emergence of therapeutic resistance within tumours.^147^, ^148^ Lactylation may potentially contribute to this process. A more comprehensive understanding of these mechanisms could pave the way for the development of strategies aimed at overcoming or preventing resistance, such as combination therapies that target both cancer cells and their lactylation pathways.

Neuroendocrine differentiation is a significant cause of resistance in lung adenocarcinoma and prostate cancer.^149^, ^150^, ^151^ Elevated global lactylation levels have been shown to drive neuroendocrine differentiation in prostate cancer.^152^ Activation of the Numb/Parkin pathway in prostate or lung adenocarcinoma leads to metabolic reprogramming of tumour cells, directly resulting in increased glycolysis and lactate levels. This, in turn, upregulates histone lactylation and promotes the expression of neuroendocrine‐related genes. Therefore, inhibitors targeting this pathway and its downstream lactylation modifications hold promise for reducing resistance in these malignancies.^58^ Patients with brain metastases from lung cancer exhibit high resistance to pemetrexed. Recent studies suggest that the AKR1B10/glycolysis/H4K12la/CCNB1 pathway is at least partly involved in the resistance of these patients. Inhibiting this pathway could be a potential strategy to combat resistance in this aggressive cancer type.^153^ Lysine lactylation partially explains the upregulation of carcinoembryonic antigen‐related cell adhesion molecule‐6 (CEACAM6) in a lactate‐rich tumour microenvironment. CEACAM6 ultimately regulates tumour cell growth, leading to resistance to 5‐fluorouracil.^154^ Diapause, typically a phenomenon where embryonic stem cells halt growth under adverse conditions,^155^, ^156^ has recently been observed in tumour cells exposed to cytotoxic drugs.^157^, ^158^ In colorectal cancer, SMC4 drives a diapause‐like transition, increasing the expression of glycolytic enzymes, which elevates lactate levels and lactylation modifications within tumours. These modifications further drive the expression of ATP‐binding cassette transporter (ABC) transporters, making tumours less sensitive to chemotherapy.^110^ Homologous recombination repair is a mechanism for repairing DNA damage.^159^ However, in tumours, this behaviour becomes a way for cancer cells to counteract cytotoxic drugs.^160^, ^161^, ^162^ Recent research has found that an enzyme called mre11, which regulates homologous recombination repair, undergoes intense lactylation mediated by the p300 enzyme. Highly lactylated mre11 exhibits stronger DNA binding affinity, thereby promoting DNA homologous recombination repair and leading to chemotherapy resistance. The use of specific small molecule peptides targeting the lactylation of MRE11 has been shown to significantly enhance the efficacy of platinum‐based or PARP inhibitor chemotherapy drugs against tumours.^163^

### 5.3 Lactylation and tumour angiogenesis

Targeted therapy is a pivotal component of contemporary cancer treatment.^164^, ^165^ Anti‐angiogenic therapy represents a significant modality within this approach.^166^, ^167^, ^168^ Angiogenesis, the formation of new blood vessels, is indispensable for tumour growth and metastasis.^147^, ^148^, ^169^ The TME, particularly hypoxic regions, promotes angiogenesis to supply the growing tumour with nutrients and oxygen. Inhibiting lactylation modification within the tumour could potentially suppress tumour‐driven internal angiogenesis. Bevacizumab, an anti‐angiogenic therapy drug, plays a substantial role in metastatic colorectal cancer. However, some patients exhibit resistance to bevacizumab, limiting its overall efficacy.^170^ Lactylation modifications are significantly upregulated in these resistant tumour cells, increasing the expression of RUBCNL and promoting autophagosome maturation, ultimately enhancing tumour survival and proliferation. Animal studies have demonstrated that combining histone lactylation inhibitors and autophagy inhibitors can improve the efficacy of bevacizumab therapy.^111^ Sema3A, a semaphorin involved in angiogenesis, has been shown to enhance the efficacy of anti‐angiogenic therapy when its expression is restored in tumours.^171^ Evodiamine, a natural anti‐tumour compound,^172^ inhibits the lactylation modification of hypoxia inducible factor 1 subunit alpha (HIF1a) by suppressing intracellular lactate production, thereby weakening HIF1a's inhibitory function on Sema3A. The relative enhancement of Sema3A function inhibits angiogenesis within the TME.^114^

### 5.4 Lactylation in cancer stem cells

With a growing understanding of cancer stem cells, immunotargeted therapies specifically targeting these stem cells are likely to be a future avenue of development.^173^, ^174^, ^175^, ^176^ Cancer stem cells are believed to be responsible for the initiation, maintenance and post‐treatment recurrence of many cancers.^177^, ^178^ These cells often exhibit distinct molecular characteristics compared to other tumour cells, including unique lactylation patterns. Targeting these distinctive lactylation patterns could potentially provide a means to combat cancer stem cells and achieve more durable therapeutic outcomes. Recent studies have demonstrated that exogenous lactate administration can enhance the tumourigenicity of liver cancer stem cells, with H3k9la and H3k56la modification sites being associated with this tumourigenicity.^113^ Under hypoxic conditions, the lactylation modification level of β‐catenin in colon cancer cells is upregulated, subsequently activating the Wnt pathway and enhancing the stemness of tumour cells.^179^ In gliomas, the upregulated NF‐κB pathway can increase the lactylation modification of H3 proteins through the Warburg effect, promoting the expression of the non‐coding RNA LINC01127. LINC01127 cis‐regulates the expression of mitogen‐activated protein kinase kinase kinase kinase 4 (MAP4K4), activates the JNK pathway, and ultimately enhances the self‐renewal capability of glioblastoma stem cells.^112^

## 6 DISCUSSION

**FIGURE 6:**
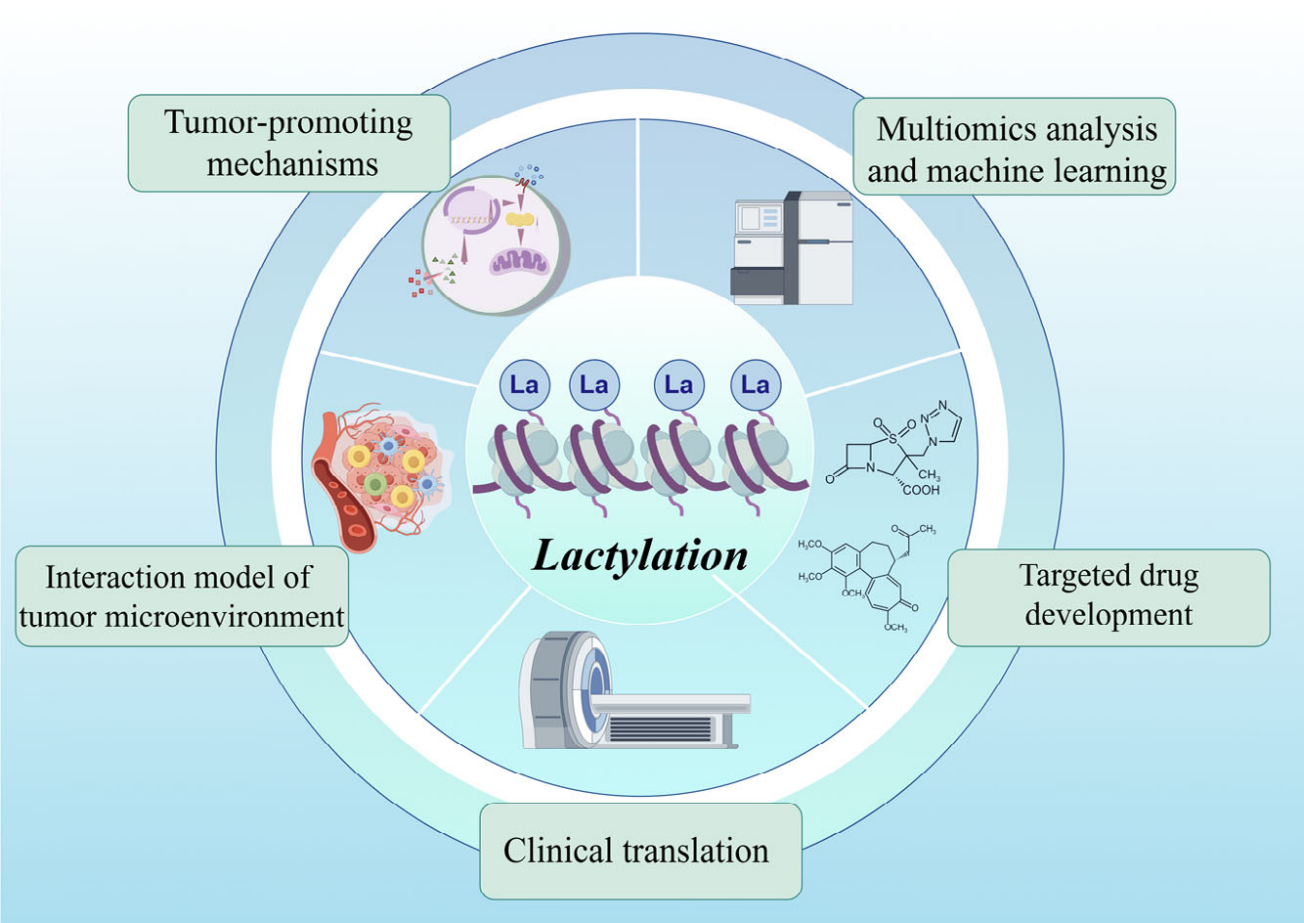
Major development directions in the field of lactylation modifications.

In summary, the current primary avenues of research within the field of lactylation modification encompass: (1) elucidating the mechanisms by which lactylation modification occurs within tumour cells; (2) investigating the role of lactylation modification in tumour microenvironment models; (3) identifying potential targets for lactylation modification through high‐throughput and machine learning approaches; (4) developing novel drugs based on these identified targets; and (5) translating this knowledge into clinical applications (Figure 6). While significant progress has been made in understanding the role of lactylation modifications in cancer, several future directions and challenges remain to be addressed by researchers.

### 6.1 Deepening the understanding of molecular mechanisms

Firstly, the mechanisms of lactylation modifications within tumour cells remain largely unexplored. Although it has been established that lactylation modifications are extensively involved in the regulation of metabolism, gene expression, and immune responses in tumour cells, the precise molecular mechanisms by which lactylation regulates specific genes and pathways – particularly the detailed mechanisms of non‐histone protein lactylation – require further investigation. These studies provide a solid theoretical foundation for designing precise drugs or intervention strategies targeting lactylation modifications.

Secondly, while the regulatory role of lactylation modifications on immune cell functions within the TME is beginning to be understood, specific mechanisms and potential therapeutic implications require further elucidation. Future research should focus on clarifying how lactylation modifications affect tumour antigen presentation, the expression of immune checkpoint molecules, and the secretion of immunosuppressive cytokines. Additionally, investigating the direct or indirect regulatory effects of lactylation modifications on the functional states of tumour‐infiltrating immune cells, such as T cells, macrophages and natural killer cells, is essential. Moreover, evaluating the correlation between lactylation modification states and immune therapy responses, as well as their potential as biomarkers for predicting immunotherapy efficacy, is crucial.

### 6.2 Discovering novel biomarkers related to lactylation modifications

Lactylation modifications have been demonstrated to correlate closely with prognosis, chemotherapy sensitivity and immunotherapy response across diverse cancer types. However, most lactylation‐related biomarkers remain in the early stages of discovery, and their clinical utility necessitates large‐scale prospective cohort studies and validation through independent datasets. Future research should prioritise the development of more sensitive, specific, and easily detectable lactylation modification biomarkers. Moreover, integrating multi‐omics data (including transcriptomics, proteomics and metabolomics) with machine learning algorithms to create comprehensive prognostic models incorporating lactylation modification information offers the potential for enhancing the accuracy of patient risk stratification, treatment selection, and efficacy prediction. Exploring changes in lactylation modifications in clinical therapies may also help identify potential biomarker targets. Future research should focus on investigating lactylation modification targets that influence tumour drug resistance and stemness. Intervening in these targets could enhance the efficacy of existing clinical therapies. Additionally, the impact of advanced and cutting‐edge treatments, such as carbon ion therapy and high‐intensity focused ultrasound (HIFU), on lactylation modifications is equally worthy of investigation.

### 6.3 Advancing the development of lactylation modification targeted therapy

Developing drugs targeting lactylation presents several challenges. One of the primary obstacles is ensuring specificity. Given the widespread occurrence of lactylation in normal physiological processes, it is essential that lactylation‐targeting drugs selectively affect cancer cells without causing undue harm to healthy tissues. Whether targeting lactylation‐modifying enzymes directly or indirectly through metabolic regulation, this issue persists. Lactylation‐modifying enzymes often share common functions with acetylation‐modifying enzymes, necessitating careful consideration of potential effects on normal intracellular acetylation levels when targeting these enzymes directly. For metabolic regulation drugs, pathways linked to glycolysis encompass a variety of biochemical activities, including mitochondrial respiration, the hexose monophosphate shunt and lipid biosynthesis. Achieving efficacy in tumour cells while minimising disruption of normal cell metabolism remains a significant challenge.

Another challenge in cancer treatment is the delivery of anticancer drugs to tumour sites. Many anticancer drugs fail to reach tumours at adequate concentrations or are rapidly metabolised by the body. Increasing blood drug concentrations can lead to adverse effects on normal tissue cells. One potential solution to address these issues is the use of emerging nanomedicine targeting carriers, which can improve drug delivery to tumour sites while minimising toxicity to healthy tissues.

### 6.4 Enhancing the interaction model between lactylation modifications and TME

Constructing a more sophisticated and comprehensive model of lactylation modification and its interplay with the tumour microenvironment is essential for a deeper understanding of how lactylation functions as a regulatory hub in driving tumour metabolic adaptation, immune evasion, and dissemination, encompassing cellular to ecosystem levels.

To achieve a comprehensive understanding of lactylation modification, modern biomedical research is increasingly integrating diverse disciplines and employing advanced techniques. Single‐cell sequencing technology enables the examination of cellular heterogeneity within the TME at the single‐cell level, revealing specific lactylation modification patterns in different cell types and their functional implications. Spatial transcriptomics provides spatial resolution at the tissue level, aiding in the identification of how lactylation modification precisely regulates gene expression within the three‐dimensional microenvironment, influencing cell‐cell interactions and microenvironmental architecture. Metabolomics studies focus on the direct effects of lactylation modification on cellular metabolic pathways, particularly glycolysis, the tricarboxylic acid cycle, and amino acid metabolism, and how these metabolic changes correlate with tumour progression and drug resistance. The inclusion of microbiomics reveals the indirect involvement of gut microbiota in TME formation and tumour progression through their modulation of host lactylation modification levels.

By integrating these multidimensional data, scientists can construct a dynamic, multi‐layered ‘lactylation modification‐tumour microenvironment’ interaction network model. This model not only elucidates the spatiotemporal distribution patterns of lactylation modification and its influence on the tumour microenvironment but also identifies novel targets and therapeutic strategies for precision medicine.

## AUTHOR CONTRIBUTIONS

**Yipeng He**: Drafted the manuscript and prepared Figures 1, 2, 3, 4, 5, 6. **Tianbao Song**: Conceived the study; supervised the research and reviewed the manuscript. **Jinzhuo Ning**; **Zefeng Wang**; **Zhen Yin** and **Qin Yuan**: Provided critical review of the manuscript. **Weimin Yu** and **Fan Cheng**: Served as corresponding authors; overseeing the research and providing a final review of the manuscript.

## CONFLICT OF INTEREST STATEMENT

The authors declare no competing interests.

## ETHICS STATEMENT

Not applicable for this review.

## Data Availability

No datasets were generated or analysed during the current study.
